# A novel role of lysophosphatidic acid (LPA) in human myeloma resistance to proteasome inhibitors

**DOI:** 10.1186/s13045-022-01269-5

**Published:** 2022-05-07

**Authors:** Pan Su, Liuling Xiao, Lingqun Ye, Zhuo Wang, Wei Xiong, Qiang Wang, Xingzhe Ma, Miao Xian, Maojie Yang, Youli Zu, Sai Ravi Pingali, Jianfei Qian, Qing Yi

**Affiliations:** 1 Center for Translational Research in Hematological Malignancies, Houston Methodist Cancer Center/Houston Methodist Research Institute, Houston, TX USA; 2 Department of Pathology and Genomic Medicine, Institute for Academic Medicine, Houston Methodist Research Institute, Houston, TX USA; 3 Houston Methodist Cancer Center, Houston Methodist Hospital, Houston, TX USA

**Keywords:** LPA, LPAR2, Multiple myeloma, Proteasome inhibitor, Drug resistance

## Abstract

Lysophosphatidic acid (LPA) is a naturally occurring phospholipid that regulates cell proliferation, survival, and migration. However, its role on human multiple myeloma (MM) cells is largely unknown. In this study, we show that LPA, which is highly elevated in MM patients, plays an important role in protecting human MM cells against proteasome inhibitor (PI)-induced apoptosis. LPA bound to its receptor LPAR2 activated its downstream MEK1/2-ERK1/2 signaling pathway and enhanced oxidative phosphorylation (OXPHOS) in mitochondria in MM cells. Increased OXPHOS activity produced more NAD^+^ and ATP, reduced proteasome activity, and enhanced protein folding and refolding in endoplasmic reticulum (ER), leading to induction of MM resistance to PIs. Importantly, inhibiting LPAR2 activity or knocking out LPAR2 in MM cells significantly enhanced MM sensitivity to PI-induced apoptosis in vitro and in vivo. Interestingly, primary MM cells from LPA-high patients were more resistant to PI-induced apoptosis than MM cells from LPA-low patients. Thus, our study indicates that LPA-LPAR2-mediated signaling pathways play an important role in MM sensitivity to PIs and targeting LPA or LPAR2 may potentially be used to (re)sensitize patients to PI-based therapy.

**Supplementary Information:**

The online version contains supplementary material available at 10.1186/s13045-022-01269-5.

To the Editor,

Multiple myeloma (MM) is a hematological malignancy that remains largely incurable and most patients relapse after one or more treatment regimens [1, 2]. The therapeutics currently available improve patient survival and quality of life, but resistance to therapy and disease progression remain unsolved issues [3]. Proteasome inhibitors (PIs) have been used as the frontline therapies for newly diagnosed and relapsed or refractory MM patients for the last two decades [4, 5]. Although PIs have shown encouraging therapeutic results, primary and secondary drug resistances and relapse after long-term treatment are inevitable in most treated patients. LPA is a naturally occurring phospholipid that regulates cell proliferation [6], survival [7] and migration [8] and exerts its effects on target cells by binding to G protein-coupled receptors (GPCRs), including LPAR1-6 [9]. Herein, we explored the mechanism underlying the regulation of LPA/LPAR2 axis on MM resistance to PI-induced apoptosis in vitro and in vivo.

MM patients (Fig. 1a) and MM-bearing mice (Fig. 1b) produced high levels of circulating LPA than their healthy controls. Moreover, primary MM cells from LPA-high patients were more resistant to PI-induced apoptosis than MM cells from LPA-low patients (Fig. 1c), and LPA treatment significantly decreased apoptosis of human primary MM cells (Fig. 1d) and cell lines (Additional file 1: Fig. S1a) induced by bortezomib (BTZ) or carfilzomib (CFZ), but not melphalan, dexamethasone, or pomalidomide (Additional file 1: Fig. S1b). Consistent with MM data in Oncomine (Fig. 1e–f), only LPAR2 among 6 LPA receptors was highly expressed in human primary MM cells (Fig. 1g) and cell lines (Additional file 1: Fig. S1c). These results suggested that the effect of LPA on MM may be derived by LPAR2. Indeed, inhibiting LPAR2 activity (Additional file 1: Fig. S1d) or knocking out LPAR2 (Fig. 1h) abolished LPA-induced apoptosis resistance in BTZ- or CFZ-treated MM cells. Further mechanistic study showed that LPA enhanced the phosphorylation of MEK1/2 and ERK1/2 only in Ctr- but not in LPAR2-KO MM cells (Fig. 1i, j, Additional file 1: Fig. S1e–S1f), and inhibiting MEK1/2-ERK1/2 signal cascade significantly attenuated the protective effect of LPA on BTZ- or CFZ-induced apoptosis in MM cells (Fig. 1k, Additional file 1: Fig. S1g).

**Fig. 1:**
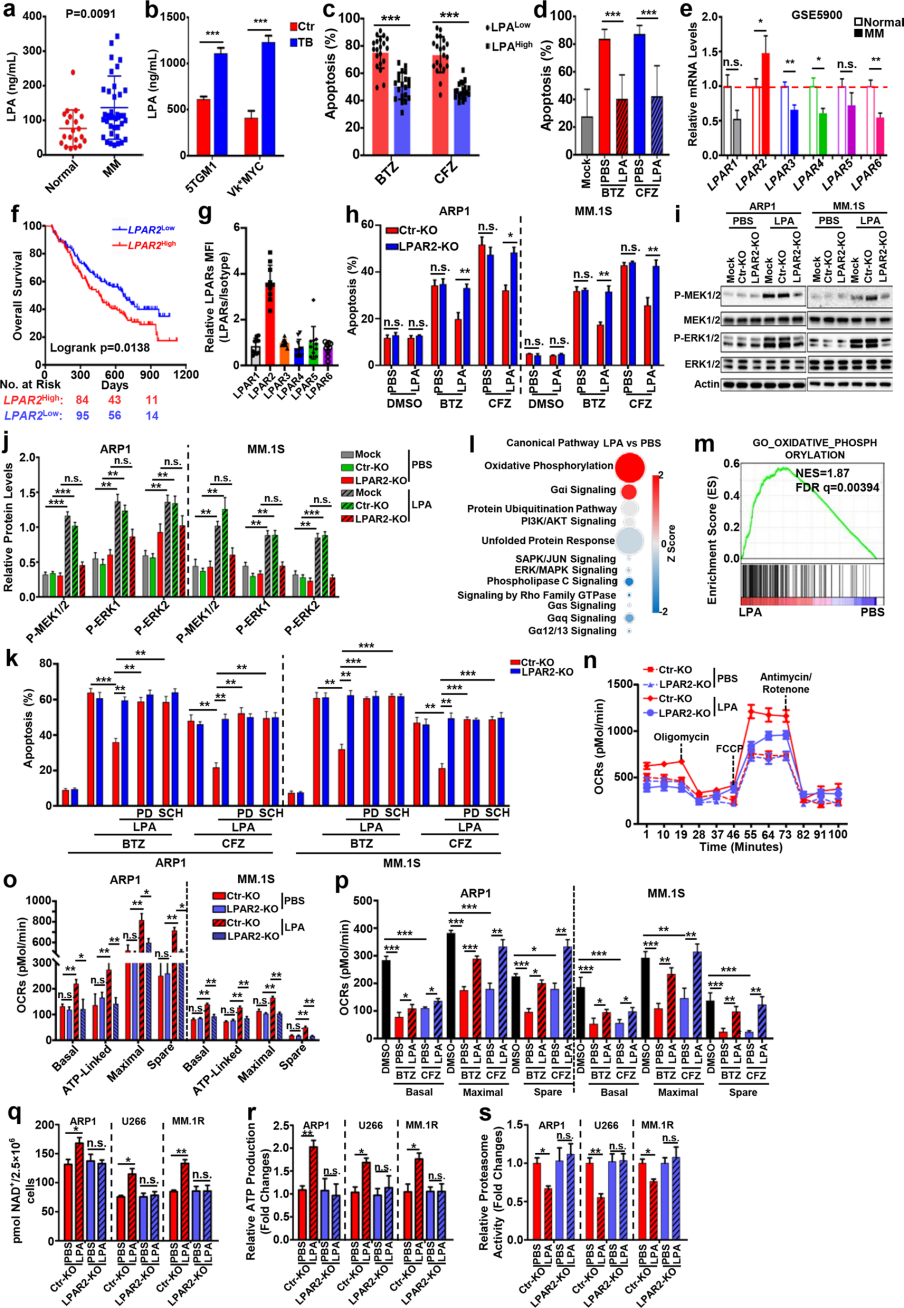
LPA enhances MM cell resistance to PIs through LPAR2-mediated MEK1/2-ERK1/2 signal pathways and enhanced OXPHOS in mitochondria. **a** Levels of LPA in serum of normal healthy controls and MM patients. **b** Levels of LPA in serum of 5TGM1 or Vk*MYC MM-tumor free (Ctr) and tumor bearing (TB) mice. Ctr, control, MM-tumor free mice; TB: MM-tumor-bearing mice. **c** The primary MM cells isolated from BM of MM patients (*n* = 20) were divided into LPA^low^ and LPA^high^ groups based on their serum levels of LPA and treated with BTZ or CFZ for one hour, then the apoptosis was measured after 24-h incubation. **d** Human primary MM cells isolated from BM of MM patients were treated with BTZ or CFZ for one hour, after wash and 24-h incubation with or without 4 μg/mL LPA, the apoptosis of the cells was determined. **e** Relative mRNA expression of LPA receptors in CD138^+^ cells of MM patients and plasma cells of normal healthy controls from GSE5900 array data. Values were normalized with normal healthy controls. **f** Overall survival of MM patients with high (*LPAR2*^High^) or low (*LPAR2*^Low^) LPAR2 expression based on published Oncomine data (GSE9782). **g** Surface expression of different LPA receptors on human primary MM cells isolated from BM of MM patients (*n* = 10). **h** Ctr-KO and LPAR2-KO ARP1 or MM.1S cells were pulsed with BTZ or CFZ for 1-h and then incubated with or without 4 μg/mL LPA for 24 h. The apoptosis of the cells were determined. Ctr-KO, MM cells transfected with lentivirus containing empty vector; LPAR2-KO, MM cells transfected with lentivirus containing LPAR2 sgRNA. **i**, **j** Ctr-KO and LPAR2-KO ARP1 or MM.1S cells were treated without (PBS) or with 4 μg/mL LPA and phosphorylation level of MEK1/2 and ERK1/2 was determined by western blot. Mock, MM cells without treatment; Ctr-KO, MM cells transfected with lentivirus containing empty vector; LPAR2-KO, MM cells transfected lentivirus containing LPAR2 sgRNA. **k** Ctr-KO and LPAR2-KO ARP1 or MM.1S MM cells were pulsed with BTZ or CFZ for one hour, followed by wash and culture with kinase inhibitors PD184352 (PD, 5 μM) or SCH772984 (SCH, 20 μM) for 24 h in the present/absent 4 μg/mL LPA, then the apoptotic rates were determined. **l** IPA analysis of canonical signaling pathway in MM cells treated without (PBS) or with 4 μg/mL LPA. The circle surface area is proportional to -log (*P* value) and the color intensity of circles indicates the *Z* score. **m** GSEA result of GO_OXIDATIVE_PHOSPHORYLATION gene signatures. NES, normalized enrichment score; FDR, false discovery rate. **n**, **o** OCRs of Ctr-KO and LPAR2-KO ARP1 cells treated with or without LPA (**n**) and summarized result of the basal respiration, ATP-linked respiration, maximal respiration, and spare capacity for Ctr-KO and LPAR2-KO ARP1 and MM.1S cells treated with or without LPA (**o**). **p** ARP1 and MM.1S cells were pulsed with BTZ or CFZ for 1 h and the cells were washed and cultured with or without LPA for another 24 h. Representative summarized results of the basal respiration, maximal respiration, and spare capacity for ARP1 and MM.1S cells. **q–s** Relative production of NAD^+^ (**q**), ATP (**r**), and the relative proteasome activity (**s**) of Ctr-KO and LPAR2-KO ARP1, U266, and MM.1R cells treated with vehicle (PBS) or LPA (4 μg/mL) for 24 h. Results are shown as means ± S.E.M.. The survival rate was analyzed by log-rank (Mantel–Cox) test. *P < 0.05; **P < 0.01; ***P < 0.001; n.s., not significant

To elucidate the molecular mechanisms downstream of LPA-LPAR2-MEK1/2-ERK1/2 pathway, Ingenuity Pathway Analysis (IPA) (Fig. 1l) and gene set enrichment analysis (GSEA) (Fig. 1m, Additional file 1: Fig. S2a–S2b) were used. Results showed that LPA significantly increased mitochondrial oxygen consumption rates (OCRs) in MM cells (Additional file 1: Fig. S2c–S2d) and this increase was impaired when LPAR2 was absent (Fig. 1n–o) or MEK1/2-ERK1/2 signal was deficient (Additional file 1: Fig. S2e–S2g). Additionally, mitochondrial respiration inhibited by BTZ or CFZ was restored by LPA treatment (Fig. 1p, Additional file 1: Fig. S2h–S2i) along with increased production of NAD^+^ (Fig. 1q) and ATP (Fig. 1r) and reduced activity of 26S proteasome (Fig. 1s) due to disruption of NAD^+^/DADH balance consistent with our GSEA analysis (Additional file 1: Fig. S2j–S2k) and a previous report [10]. As enhanced OXPHOS and ATP production are  involved in ER protein folding/refolding essential for MM cell survival [11], we further analyzed this process in MM cells. Results showed that genes involved in ER protein folding/refolding were highly expressed in MM cells of patients compared to normal plasma cells (Fig. 2a, Additional file 1: Fig. S3a) and positively correlated with the level of LPAR2 in MM patients (Fig. 2b, Additional file 1: Fig. S3b). ER protein folding/refolding ability (Fig. 2c) and ER ATP distribution (Fig. 2d–e) were increased in LPA-treated MM cells. ER retained relatively high ROS levels due to protein folding processes (Additional file 1: Fig. S3c–S3f) and ROS assays (Additional file 2) [12] showed that the capacity of MM cells to buffer the formation of ROS was enhanced by LPA (Fig. 2f, Additional file 1: Fig. S3g). Suppression of MEK1/2-ERK1/2 signal by PD184352 and SCH772984 abolished this buffer capacity (Fig. 2f, Additional file 1: Fig. S3g), further suggesting the dependence of LPA function on MEK1/2-ERK1/2 pathway.

**Fig. 2:**
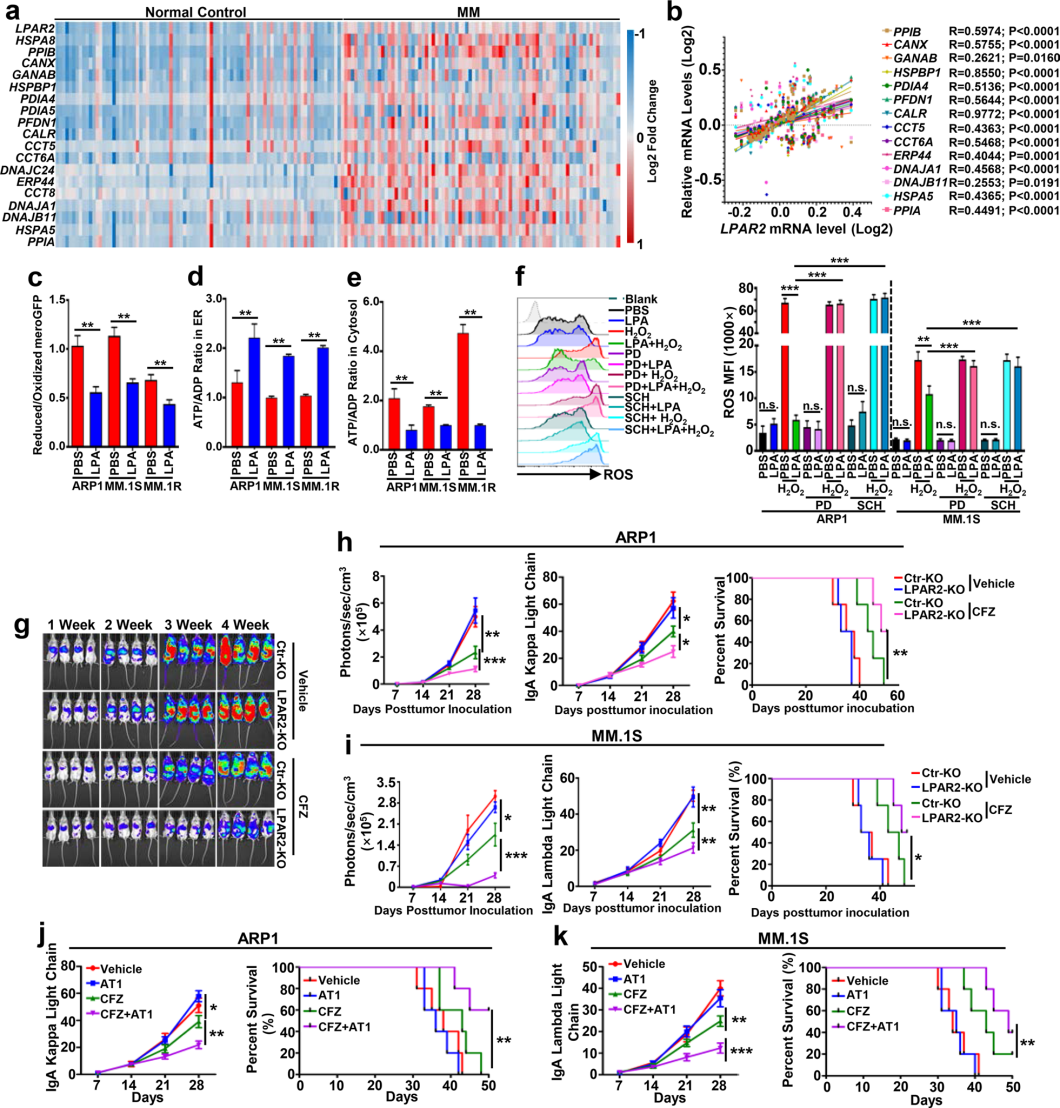
LPAR2 deficiency or inhibition sensitizes human MM cells to PI treatment through regulating mitochondrial OXPHOS-mediated ER protein folding/refolding and proteasome activity. **a** Heatmap showing the relative expression of *LPAR2* and genes involved in protein folding/refolding in ER in normal plasma cells and patient-derived MM cells from GSE15695. **b** Correlations between *LPAR2* and gene cluster involved in protein fold/refolding in ER, including *PPIB*, *CANX*, *GANAB*, *HSPBP1*, *PIAD4*, *PFDN1*, *CALR*, *CCT5*, *CCT6A*, *ERP44*, *DNAJA1*, *DNAJB11*, *HSPA5*, and *PPIA*, in patient-derived MM cells from GSE15695. **c–e** Bar graphs depicting the summarized results of the reduced/oxidized meroGFP in ER (**c**), the ATP/ADP ratio in ER (**d**) and cytosol (**e**) of ARP1, MM.1S, and MM.1R cells treated without (PBS) or with 4 μg/mL LPA. **f** ARP1 and MM.1S cells were pre-treated with vehicle (PBS), LPA (4 μg/mL), vehicle + PD184352 (5 μM), LPA + PD, vehicle + SCH772984 (20 μM), or LPA + SCH for 24 h followed with or without 30-min H_2_O_2_ (0.08%) treatment, then the ROS levels were measured. **g**, **h** NSG mice were injected i.v. with 2 × 10^6^ Ctr-KO or LPAR2-KO ARP1-luc MM cells. On day 7 after tumor inoculation, vehicle or 3 mg/kg CFZ were i.p. injected for 2 consecutive days in a week and repeated for 3 weeks. Tumor burden measured by bioluminescent imaging (**g** and left panel of **h**) and serum concentration of IgA kappa light chain (middle panel of **h**) and survival (right panel of **h**) were showed. **i** NSG mice were injected i.v. with 2 × 10^6^ Ctr-KO or LPAR2-KO MM.1S-luc MM cells and treated as above. Summarized results showing tumor burden measured as bioluminescent images (left panel of **i**) and serum concentration of IgA lambda light chain (middle panel of **i**) and survival (right panel of **i**) of indicated mice. **j**, **k** NSG mice were injected i.v. with 2 × 10^6^ ARP1 (**j**) or MM.1S (**k**) MM cells. On day 7 after tumor inoculation, vehicle, AT1 (0.2 mg/kg), CFZ (3 mg/kg) or CFZ + AT1 were i.p. injected for 2 consecutive days in a week and repeated for 3 weeks. Tumor burdens (left panels) and survival (right panels) were monitored. Results are shown as means ± S.E.M. *P < 0.05; **P < 0.01; ***P < 0.001; n.s., not significant

To further investigate the translational potential of targeting LPAR2-mediated signal cascade to overcome MM cell PI resistance in vivo, we examined the therapeutic effect of CFZ on human Ctr- or LPAR2-KO ARP1- (Fig. 2g, h) or MM.1S-Luc (Fig. 2i, Additional file 1: Fig. S3h) bearing mice. Consistent with in vitro results, CFZ treatment resulted in significantly smaller tumor burdens and prolonged survival in mice bearing LAPR2-KO MM cells compared to Ctr-KO MM cells (Fig. 2g–i, Additional file 1: Fig. S3h). Similarly, combination of CFZ with LPAR2 inhibitor dramatically reduced tumor burden and prolonged mouse survival compared to CFZ alone (Fig. 2j, k), indicating the translational potential of targeting LPAR2-mediated signal cascade to overcome MM cell PI resistance in vivo.

In summary, we described a novel mechanism underlying the induction of MM resistance to PI-induced apoptosis. Our findings may not only contribute to a better understanding of the importance of the bioactive lipid in MM resistance of PIs but also highlight the importance of targeting LPA-LPAR2-mediated signaling pathway as a potential therapeutic approach to overcome MM resistance to PI treatment in patients.

## Supplementary Information

**Additional file 1.** Supplementary Figures and Figure legends.

**Additional file 2.** Supplementary Materials and Methods.

## Data Availability

Datasets used and/or analyzed during the current study are available from the corresponding author on reasonable request.

## Abbreviations

LPA: Lysophosphatidic acid
MM: Multiple myeloma
PI: Proteasome inhibitor
OXPHOS: Oxidative phosphorylation
GPCRs: G protein-coupled receptors
BTZ: Bortezomib
CFZ: Carfilzomib
OCR: Oxygen consumption rate
ER: Endoplasmic reticulum

## References

[1] Pinto V, Bergantim R, Caires HR, Seca H, Guimaraes JE, Vasconcelos MH (2020). Multiple myeloma: available therapies and causes of drug resistance. Cancers (Basel).

[2] Hong S, Qian J, Yang J, Li H, Kwak LW, Yi Q (2008). Roles of idiotype-specific t cells in myeloma cell growth and survival: Th1 and CTL cells are tumoricidal while Th2 cells promote tumor growth. Cancer Res.

[3] Wang Q, Lin Z, Wang Z, Ye L, Xian M, Xiao L, Su P, Bi E, Huang YH, Qian J (2022). RARgamma activation sensitizes human myeloma cells to carfilzomib treatment through the OAS-RNase L innate immune pathway. Blood.

[4] Ito S (2020). Proteasome inhibitors for the treatment of multiple myeloma. Cancers (Basel).

[5] Leleu X, Martin TG, Einsele H, Lyons RM, Durie BGM, Iskander KS, Ailawadhi S (2019). Role of proteasome inhibitors in relapsed and/or refractory multiple myeloma. Clin Lymphoma Myeloma Leuk.

[6] Kim D, Li HY, Lee JH, Oh YS, Jun HS (2019). Lysophosphatidic acid increases mesangial cell proliferation in models of diabetic nephropathy via Rac1/MAPK/KLF5 signaling. Exp Mol Med.

[7] Kostic I, Fidalgo-Carvalho I, Aday S, Vazao H, Carvalheiro T, Graos M, Duarte A, Cardoso C, Goncalves L, Carvalho L (2015). Lysophosphatidic acid enhances survival of human CD34(+) cells in ischemic conditions. Sci Rep.

[8] Brusevold IJ, Tveteraas IH, Aasrum M, Odegard J, Sandnes DL, Christoffersen T (2014). Role of LPAR3, PKC and EGFR in LPA-induced cell migration in oral squamous carcinoma cells. BMC Cancer.

[9] Geraldo LHM, Spohr T, Amaral RFD, Fonseca A, Garcia C, Mendes FA, Freitas C, dosSantos MF, Lima FRS (2021). Role of lysophosphatidic acid and its receptors in health and disease: novel therapeutic strategies. Signal Transduct Target Ther.

[10] Tsvetkov P, Myers N, Eliav R, Adamovich Y, Hagai T, Adler J, Navon A, Shaul Y (2014). NADH binds and stabilizes the 26S proteasomes independent of ATP. J Biol Chem.

[11] Braakman I, Helenius J, Helenius A (1992). Role of ATP and disulphide bonds during protein folding in the endoplasmic reticulum. Nature.

[12] Merksamer PI, Trusina A, Papa FR (2008). Real-time redox measurements during endoplasmic reticulum stress reveal interlinked protein folding functions. Cell.

