# Supplementary material for: A novel role of lysophosphatidic acid (LPA) in human myeloma resistance to proteasome inhibitors
Source: J Hematol Oncol. 2022 May 7;15:55. doi: 10.1186/s13045-022-01269-5 (PMC9077919; doi:10.1186/s13045-022-01269-5)
Supplement: Supplementary file 1 — Additional file 1. Supplementary Figures and Figure legends. [file 13045_2022_1269_MOESM1_ESM.docx]

**Supplementary Figures and Figure legends**

**Fig. S1**

**
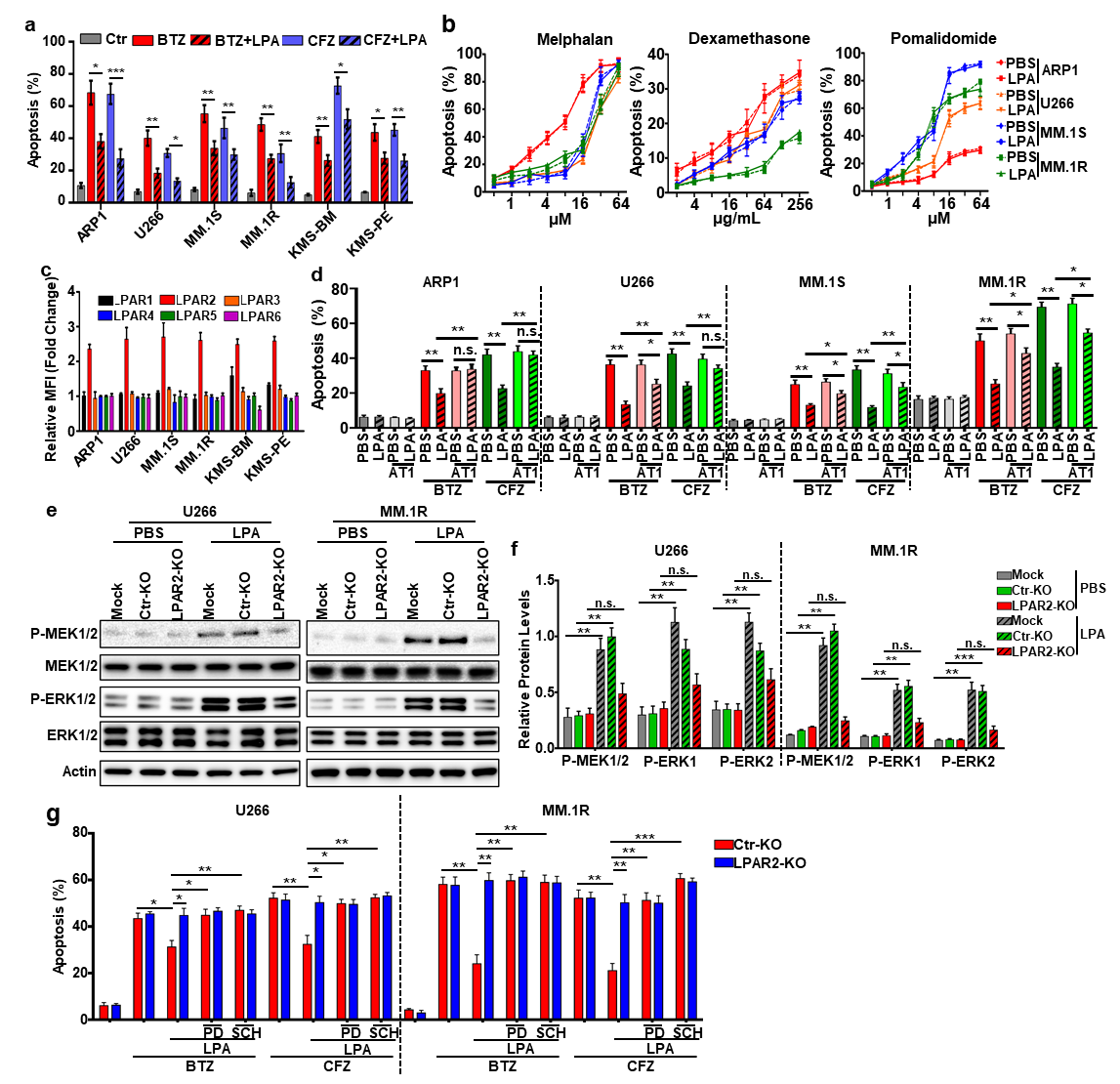
**

**Fig. S1. LPA regulates the sensitivity of MM cells to PIs through LPA/LPAR2-mediated activation of MEK1/2-ERK1/2 pathway.**

(**a**) Human ARP1, U266, MM.1S, MM.1R, KMS-BM, and KMS-PE MM cells were treated with BTZ (100 nM) or CFZ (80 nM) for one hour and followed by wash and incubation with or without 4 μg/mL LPA treatment for 24 hours. Representative summarized result showing apoptosis of human ARP1, U266, MM.1S, MM.1R, KMS-BM, and KMS-PE MM cells. (**b**) Summarized results showing apoptosis of ARP1, U266, MM.1S, and MM.1R cells in culture with different concentrations of melphalan, dexamethasone, or pomalidomide without (PBS) or with LPA (4 μg/mL). (**c**) Bar graph showing surface expression of indicated LPA receptors on different MM cell lines. (**d**) Bar graph depicting apoptosis of ARP1, U266, MM.1S, and MM.1R MM cells treated with pulse treatment of BTZ (100 nM) or CFZ (80 nM) for one hour, followed by wash and culture with vehicle (PBS), LPA (4 μg/mL), vehicle + AT1 (5 μM), and LPA + AT1 for 24 hours. (**e, f**) Western blot (**e**) showing phosphorylated MEK1/2 and ERK1/2 in Mock, Ctr-KO or LPAR2-KO U266 and MM.1R cells treated without (PBS) or with 4 μg/mL LPA. Bar graph (**f**) representing normalized values of phosphorylated MEK1/2 or ERK1/2 to total MEK1/2 or ERK1/2 respectively. Mock, MM cells without treatment; Ctr-KO, MM cells transfected with lentivirus containing empty vector; LPAR2-KO, MM cells transfected with lentivirus containing LPAR2 sgRNA. (**g**) Summarized result showing apoptosis of Ctr-KO and LPAR2-KO U266 and MM.1R cells in one-hour pulse with 100 nM BTZ or 80 nM CFZ, followed by wash and incubation with vehicle (PBS), LPA (4 μg/mL), vehicle + PD (5 μM), LPA + PD, vehicle + SCH (20 μM), or LPA + SCH for 24 hours. Results are shown as means ± S.E.M. Statistical significance was determined by two-tailed Student t test between indicated groups. *P < 0.05; **P < 0.01; ***P < 0.001; n.s., not significant.

**Fig. S2**

**
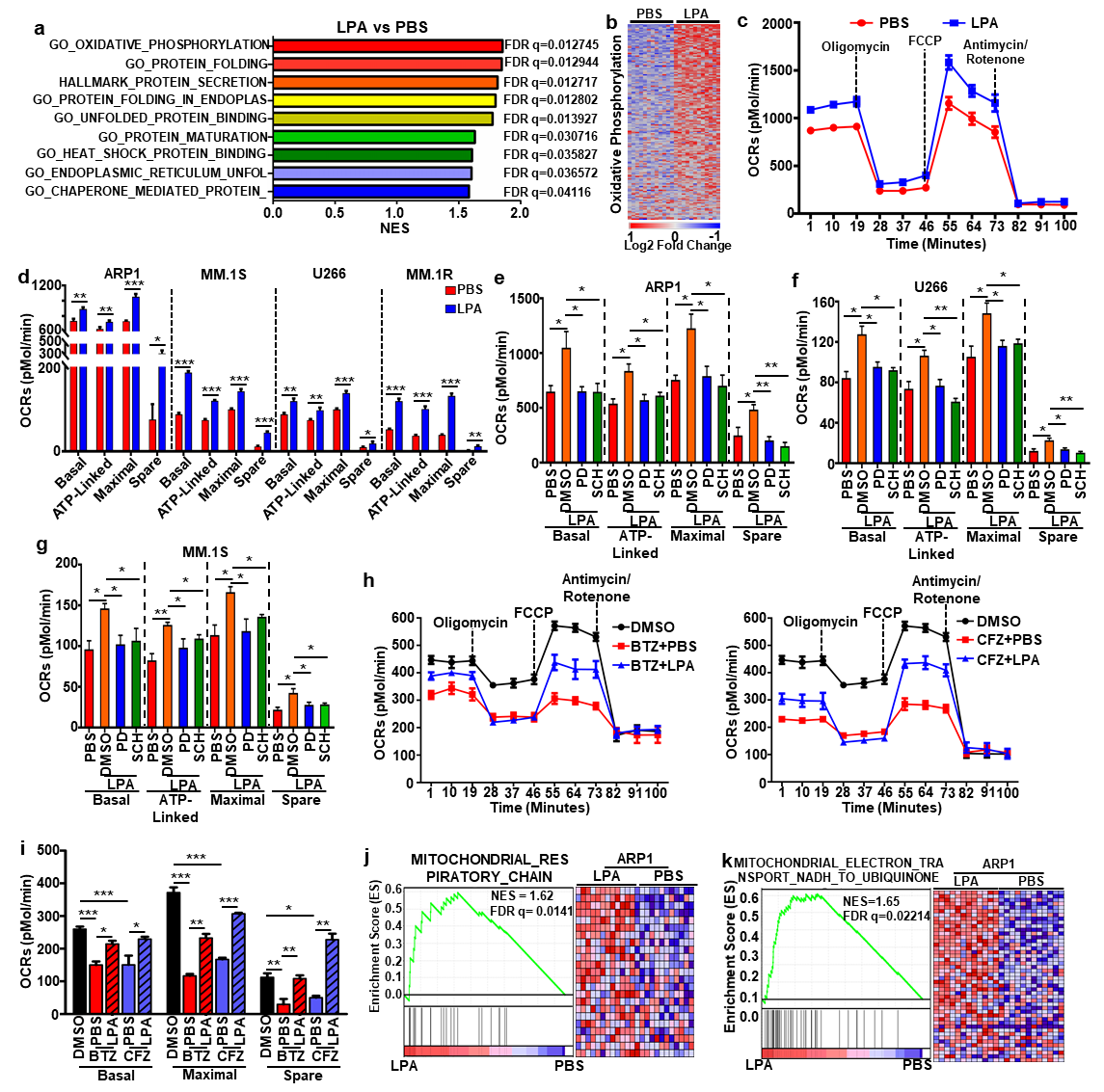
**

**Fig. S2. LPA enhances mitochondrial activity in MM cells.**

(**a**) Representative bar graph showing the summarized results of GSEA analysis. (**b**) Heatmap illustrating the relative expression of genes involved in OXPHOS. (**c, d**) OCRs of ARP1 cells treated without (PBS) or with 4 μg/mL LPA (**c**) and summarized result (**d**) of the basal respiration, ATP-linked respiration, maximal respiration, and spare capacity for ARP1, MM.1S, U266, and MM.1R cells. (**e-g**) Summarized results showing the basal respiration, ATP-linked respiration, maximal respiration, and spare capacity in ARP1 (**e**), U266 (**f**), or MM.1S (**g**) cells treated without (PBS) or with 4 μg/mL LPA in the presence or absence of PD at 5 μM or SCH at 20 μM for 24 hours. (**h, i**) U266 cells were pulsed without (DMSO) or with 100 nM BTZ or 80 nM CFZ for 1 hour, followed by wash and incubation without (PBS) or with 4 μg/mL LPA for 24 hours. Shown are OCRs in U266 cells treated with BTZ (**h**, left panel) or CFZ (**h**, right panel), and summarized results (**i**) of basal respiration, maximal respiration, and spare capacity in the cells. (**j, k**) Representative GSEA enrichment plots (left panels) and heatmaps (right panels) showing the distribution and expression of the MITOCHONDRIAL_RESPIRATORY_CHAIN (**j**) and GOBP_MITOCHONDRIAL_ELECTRON_TRANSPORT_NADH_TO_UBIQUINONE (**k**) gene signatures. NES, normalized enrichment score; FDR, false discovery rate. Results are shown as means ± S.E.M. Statistical significance was determined by two-tailed Student t test between indicated groups. *P < 0.05, **P < 0.01, ***P < 0.001.

**Fig. S3**


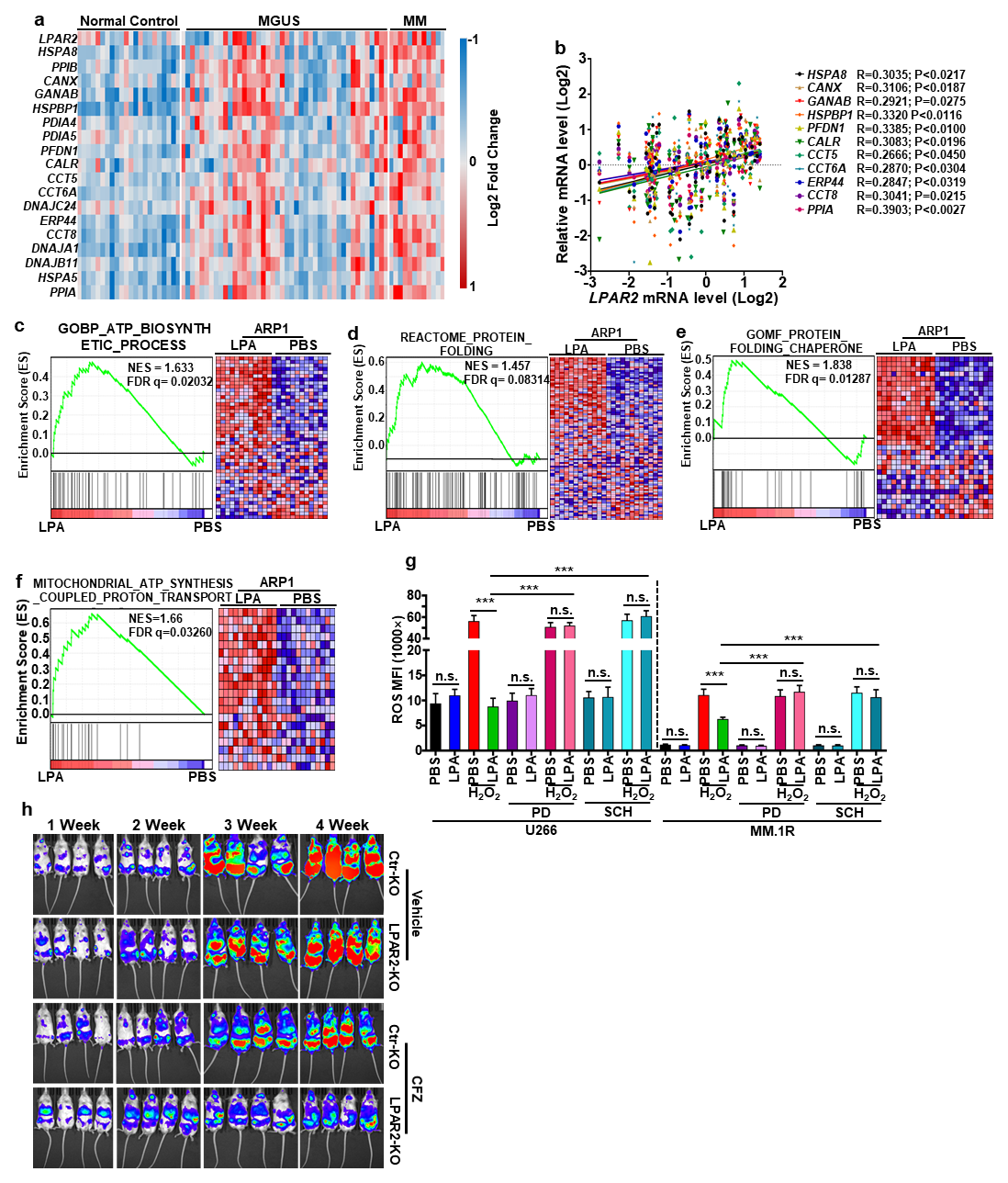


**Fig. S3. LPA promotes protein folding/refolding in ER of MM cells.**

(**a**) Heatmap showing the relative expression of *LPAR2* and genes involved in protein folding/refolding in ER in normal plasma cells and patient-derived MM cells from GSE5900. (**b**) Correlations between *LPAR2* and gene cluster involved in protein fold/refolding in ER, including *HSPA8*, *CANX*, *GANAB*, *HSPBP1*, *PFDN1*, *CALR*, *CCT5*, *CCT6A*, *ERP44*, *CCT8*, and *PPIA*, in patient-derived MM cells from GSE5900. (**c-f**) Representative GSEA enrichment plots (left panels) and heatmaps (right panels) showing the distribution and expression of the GOBP_ATP_BIOSYNTHETIC_PROCESS (**c**),

REACTOME_PROTEIN_FOLDING (**d**), GOMF_PROTEIN FOLDING CHAPERONE (**e**), and GOBP_MITOCHONDRIAL_ATP_SYNTHESIS_COUPLED_PROTON_TRANSPORT (**f**) gene signatures. NES, normalized enrichment score; FDR, false discovery rate. (**g**) U266 and MM.1R cells were pre-treated with vehicle (PBS), LPA (4 μg/mL), vehicle + PD (5 μM), LPA + PD, vehicle + SCH (20 μM), or LPA + SCH for 24 hours, followed by 30-minute H_2_O_2_ (0.08%) treatment or water. Summarized results showing ROS level in U266 and MM.1R cells. (**h)** NSG mice were injected i.v. with 2×10^6^ Ctr-KO or LPAR2-KO MM.1S-luc MM cells. On day 7 after tumor inoculation, vehicle or 3 mg/kg CFZ were i.p. injected for 2 consecutive days in a week and repeated for 3 weeks. Tumor burden was detected by bioluminescent imaging. Shown are bioluminescent images of NSG mice bearing Ctr-KO or LPAR2-KO MM.1S-luc MM cells treated without (Vehicle) or with CFZ (3 mg/kg, i.p. injection) from weeks 1 to 4 after tumor injection. Tumor burden was detected by bioluminescent imaging. Results are shown as means ± S.E.M. Statistical significance was determined by two-tailed Student t test between indicated groups. ***P < 0.001; n.s., not significant.
