# Supplementary material for: A novel role of lysophosphatidic acid (LPA) in human myeloma resistance to proteasome inhibitors
Source: J Hematol Oncol. 2022 May 7;15:55. doi: 10.1186/s13045-022-01269-5 (PMC9077919; doi:10.1186/s13045-022-01269-5)
Supplement: Supplementary file 2 — Additional file 2. Supplementary Materials and Methods. [file 13045_2022_1269_MOESM2_ESM.docx]

**Materials and Methods**

**Human Samples**

Bone marrow (BM) aspirates were collected from newly diagnosed patients with MM. BM aspirates from healthy donors were used as controls. BM CD138^+^ MM cells were isolated by EasySep™ Human Whole Blood and CD138 Positive Selection Kit II (STEMCELL Technologies, MA). All human participants provided written informed consent, and this study was conducted in accordance with the U.S. Common Rule and approved by the Institutional Review Board at Houston Methodist Research Institute.

**Mice**

C57BL/6J and NOD.Cg-*Prkdc^scid^ Il2rg^tm1Wjl^*/SzJ (NSG) mice were purchased from the Jackson laboratory. C57BL/KalwRij mice were purchased from Envigo (Horst, Netherland). All mice used in this study were maintained in a temperature- and humidity-controlled environment and given unrestricted access to chow diet and acidified water. The studies were approved by the Institutional Animal Care and Use Committee of the Houston Methodist Research Institute.

***In vivo* xenograft mouse model**

To examine the role of LPA and its receptor LPAR2 in MM response to carfilzomib (CFZ) *in vivo*, human ARP1 and MM.1S MM xenografted murine models were used. Briefly, NSG mice at 8-week of age were injected i.v. with 2 × 10^6^ ARP1-Luc-Ctr-KO/LPAR2-KO or MM.1S-Luc-Ctr-KO/LPAR2-KO cells. Seven days later, vehicle or 3 mg/kg CFZ were i.p. injected into MM-bearing mice (two consecutive days weekly for 3 weeks). To determine whether inhibition of LPAR2 pathway enhances the therapeutic efficacy of CFZ in MM *in vivo*, NSG mice were injected i.v. with 2 × 10^6^ human MM cell lines ARP1 or MM.1S. On day 7 after tumor inoculation, MM-bearing mice were injected with vehicle, LPAR2 antagonist 1 (AT1) (0.2 mg/kg for every 3 days), CFZ, and AT1 plus CFZ. Blood samples were collected weekly starting on day 7. Tumor burdens were evaluated by bioluminescence on IVIS imaging system and ELISA for determining the level of human immunoglobulin light chain secreted by human MM cells in mouse plasma, which was normalized to control. Survival was monitored, and mice were sacrificed when they develop signs of hind limb paralysis.

**Cell lines and cell culture**

Human MM cell lines ARP1, U266, MM.1S, MM.1R, RPMI 8226, KMS-BM, KMS-PE, and human Phoenix-AMPHO cells were purchased from ATCC. Murine myeloma 5TGM1 cell line was a kind gift from Dr. Frederic J Reu and in culture with IMDM medium supplemented with 10% FBS (Gibco). Vk*MYC cells were provided by Leif P. Bergsagel (Mayo Clinic in Arizona, Phoenix, Arizona, USA). All cells were cultured as described previously [1]. Briefly, cells were cultured in RPMI 1640 or DMEM medium supplemented with 10% FBS (Gibco), 100 U/ml penicillin, and 100 μg/ml streptomycin at 37 °C with 5% CO_2_. Cells were passed every 2-3 days to keep logarithmic growth. RPMI 1640 medium supplemented with 0.1% fatty-free BSA (Sigma), 100 U/ml penicillin, and 100 μg/ml streptomycin was used to culture the cells with LPA *in vitro*. Mycoplasma testing and cell authentication were performed by the Cytogenetics and Cell Authentication Core of MD Anderson Cancer Center (Houston, TX). All cells used in this study were mycoplasma negative.

**Antibodies, drugs, inhibitors, and plasmids**

Anti-LPAR1 (sc-515665) and anti-LPAR3 (sc-390270) antibodies were purchased from Santa Cruz Biotechnology. Anti-LPAR4 (MAB10217-SP) and anti-LPAR5 (MAB10297-SP) antibodies were purchased from R&D System. Anti-LPAR2 (bs-10368R) antibody was purchased from Bioss Antibodies or ThermoFisher. Anti-LPAR6 (A05725-1) antibody was purchased from Boster Company. Western blot antibodies against P-MEK1/2 (#9121), MEK1/2 (#9122), P-ERK1/2 (#9101), ERK1/2 (#4695), P-JNK (#4668), JNK (#9252), P-PI3K (#17366), PI3K (#4257), P-P38 (#4511), P38 (#8690), P-PLCg2 (#50535), PLCγ2 (#55512), P-Raf (#9423), Raf (#53745), c-Myc (#18583), and β-actin (#8457) were purchased from Cell Signaling Technology. The ERK1/2 inhibitor SCH772984 (S7101) and MEK1/2 inhibitor PD184352 (CI-1040) (S1020) were purchased from Selleckchem. LentiCRISPRv2GFP plasmid was purchased from Addgene (Plasmid #82416).

**Flow Cytometry**

Surface expression of six LPA receptors on different MM cells was determined using standard staining and flow cytometry techniques as described before [2]. Briefly, MM cells were collected and washed with ice-cold PBS, incubated with indicated antibodies in flow cytometry staining buffer for 30 minutes on ice after Fc blocking, followed by washing and incubation with indicated fluorophore-conjugated secondary antibodies for another 30 minutes at room temperature. Data were acquired on LSRFortessa (BD Biosciences) and analyzed with FlowJo V10 Software (FlowJo Company, Ashland, OR, USA).

**Measurement of LPA**

The levels of LPA in human or mice serum were determined by using the General Lysophosphatidic Acid ELISA Kit (MyBioSource, MBS2700658) according to the manufacture’s instruction. All samples were run in triplicate. The standard curve for LPA ranges from 20 μg/mL to 100 ng/mL.

**Annexin V staining assay**

Apoptosis of human MM cells was determined by using APC Annexin V Apoptosis Detection Kit (640932, BioLegend) according to the manufacturer’s instruction.

**Western blot analysis**

For immunoblot analysis, cells with different treatments were lysed using 10 × cell lysis buffer (#9803, Cell Signaling Technology) supplemented with protease and phosphatase inhibitors on ice for 10 to 30 minutes. Samples were sonicated and centrifuged at 13,000 × g for 10 minutes at 4°C to remove insoluble materials. Protein concentration was determined using the Coomassie Protein Assay Reagent (Prod# 1856209, ThermoFisher). Equal amounts (20 μg) of protein were loaded on SDS-PAGE and transferred to polyvinylidene difluoride membrane, followed by blocking membranes with 5% milk and incubation with indicated primary antibodies overnight at 4°C and then, after washing, with HRP-conjugated secondary antibodies for one hour. Imaging of western blots was performed using chemiluminescent ECL substrate on ChemiDoc^TM^ MP Imaging System (BioRad).

**Total mRNA sequencing**

For RNA-seq analysis, total RNA was isolated from ARP1 cells cultured with or without LPA by using TRIzol RNA isolation reagents (15596026, Invitrogen^TM^). Quality control analysis was performed using Agilent 2100 Bioanalyzer (Agilent). RNA-seq was performed at the Cancer Genomics Center of The University of Texas Health Science Center in Houston. Raw data were normalized and analyzed for differential expression in R using the Bioconductor package DEseq2. For Gene Set Enrichment Analysis (GSEA), GSEA was run for each sample in pre-ranked list mode with 1000 permutations (normal P-value cutoff < 0.05). The gene sets from the Broad Institute Molecular Signature Database were used:

GO_OXIDATIVE_PHOSPHORYLATION

GO_PROTEIN FOLDING

HALLMARK_PROTEIN_SECRETION

GO_PROTEIN_FOLDING_IN_ENDOPLASMIC_RETICULUM

GO_UNFOLDED_PROTEIN_BINDING

GO_PROTEIN_MATURATION

GO_HEAT_SHOCK_PROTEIN_BINDING

GO_ENDOPLASMIC_RETICULUM_UNFOLDED_PROTEIN_RESPONSE

GO_CHAPERONE_MEDIATED_PROTEIN_FOLDING

DESeq2 results were input into IPA for further pathway enrichment analysis (https://www.qiagenbioinformatics.com/products/ ingenuitypathway-analysis).

**PI treatment**

To mimic *in vivo* patient pharmacokinetics of PIs, human MM cells were pulsed with high dose of PIs bortezomib (BTZ) (100 nM) or CFZ (80 nM) for one hour and recovered in PI-free medium (0.1% fatty-free BSA contained RPMI 1640) for another 24 hours.

**Seahorse Assay**

The seahorse XFe96 Extracellular Flux Analyzer (Seahorse Bioscience) was used to measure OCRs in MM cells. Briefly, Ctr-KO or LPAR2-KO MM cells were pretreated with or without indicated reagents for 24 hours and then transferred into a pre-Cell-Tak-coated XFe96-well plate (3 × 10^5^/well). Cells were washed and incubated in Seahorse XF RPMI Medium (Cat. #103576-100, Agilent) supplemented with 10 mM glucose (Cat. #103577-100, Agilent), 1 mM sodium pyruvate (Cat. #103578-100, Agilent), and 2 mM glutamine (Cat. #103579-100, Agilent) in a non-CO_2_ incubator at 37°C for 20 minutes. Oligomycin (1 µM), FCCP (1.5 µM) and rotenone (0.5 µM) were sequentially injected as indicated and OCR was recorded in real time.

**Measurement of NAD^+^/NADH ratio**

NAD^+^/NADH ratio in treated MM cells was determined by using a NAD^+^/NADH Quantitation Colorimetric Kit (K337, BioVision) according to the manufacturer’s instruction. Briefly, MM cells with or without indicated treatment were washed with ice-cold PBS and lysed with NAD^+^/NADH Extraction Buffer (K337-100-1, BioVision) by freeze/thaw for two cycles (20 minutes on dry-ice, then 10 minutes at room temperature), and then the samples were vortexed and centrifuged at 13,000 × g for 5 minutes to collect extracted NAD^+^/NADH supernatant. For each of the extracted samples, half of the sample was heated to 60°C for 30 minutes to decompose NAD^+^ while keeping NADH intact to extract NAD^+^ (NAD^+^ + NADH). Both NADt (NAD total) and NADH samples were gently mixed with NAD cycling enzyme and absorbance was measured at 450 nm using a CLARIOstar Plate Reader (BMG LABTECH). NADH standard curve was used to quantify NADt and NADH, and the results were normalized to pmol/2.5 × 10^6^

cells.

**Determination of proteasome activity**

Proteasome activity of treated MM cells was determined with Proteasome Activity Assay Kit (ab107921, Abcam) according to the manufacturer’s protocol. Briefly, MM cells (2 × 10^6^ per well) seeded in 6-well plates were washed with 500 μL ice-cold PBS and homogenized with 0.5% NP-40 buffer by pipetting up and down several times. Samples were centrifuged at 13,000 × g at 4°C for 10-15 minutes to remove insoluble materials. Generated cytoplasmic supernatant was used for determination of proteasome activity. Fluorescence produced by the cleavage of 7-amino-4-methylcoumarin (AMC)-labeled substrate was measured using a CLARIOstar Plate Reader at excitation/emission = 350/460 nm. Proteasome inhibitor was added to cytoplasmic supernatant for negative controls.

**Determination of intracellular ROS buffering capacity**

MM cells pre-treated with or without LPA were incubated with 2 μM CM-H2DCFDA (C6827, ThermoFisher) or CellROX™ Deep Red Reagent (C10422, ThermoFisher) as a general oxidative stress indicator at 37°C for 20 minutes. H_2_O_2_ (0.08%) was added to the cells and incubated for 30 minutes at 37°C. After incubation, cells were washed, and green fluorescence intensity was determined with LSRFortessa (BD Biosciences). Data were analyzed using FlowJo V10 Software (FlowJo Company, Ashland, OR, USA).

**Transfection of MM cells with reporter plasmids**

The transduction of MM cells with reporter plasmids was performed as previously described [3]. MM cells (5 × 10^5^ cells) were transfected with indicated reporter plasmids (2 μg) using the 4D-Nucleofector^TM^ System (Lonza) in 82 μL SF Cell Line Nucleofector^TM^ Solution supplemented with 18 μL Supplement 1. For reporter plasmids with puromycin resistance, a brief puromycin selection (0.5 μg/mL) was performed on transfected cells for one week. Surviving cells were allowed to recover and expand for two weeks before the assay.

The plasmid GW1-PercevalHR (#49082) was purchased from Addgene. The plasmid ERAT4.01 was purchased from Next Generation Fluorescence Imaging Company (NGFI, Austria). The full sequences of MERO-GFP were kindly provided by Dr. Fumihiko Urano [4] and Dr. Kohsuke Kanekura.

**Analysis of ATP/ADP ratio in cytosol and ER**

ATP/ADP ratio in cytosol or ER of treated MM cells was determined using a genetically encoded sensor GW1-PercevalHR plasmid as described previously [3]. Briefly, the fluorescence intensity of indicated channels in treated MM cells was recorded using an LSRFortessa flow cytometer. Cytosolic ATP/ADP ratio for individual cells was defined as the ratio of fluorescence intensity of channel B (Ex/Em: 488/510 nm) divided by that of channel A (Ex/Em: 405/525 nm). A pHrodo™ Red AM Intracellular pH Indicator (P35372, ThermoFisher) was used to determine pH changes in cells. ER ATP level was defined as the ratio of fluorescence intensity of channel B (Ex/Em: 405/530) divided by that of channel A (Ex/Em: 405/445).

**Determination of ER redox state**

To determine the redox state in ER, a MERO-GFP sensor system was used [5]. Briefly, treated MM cells were seeded into 96-well plates and fluorescence at excitation 473 nm and emission 510 nm (for reduced MERO-GFP) or at excitation 394 nm and emission 510 nm (for oxidized MERO-GFP) was reordered. MERO-GFP ratio was determined after subtraction of background signal.

**Statistics**

Protein band quantification was analyzed by the ImageJ software (NIH). To compare various experimental groups, the student *t* test (two-tailed) was used. When more than two groups were included in an analysis, the Bonferroni’s corrected significance level was used. The Kaplan-Meier estimates and log-rank tests were used to evaluate the survival rates. The difference of tumor volumes was analyzed using one-way AVOVA. All results in this study are shown as mean ± S.E.M., unless otherwise indicated. A *P* value less than 0.05 was considered statistically significant.

**References:**

1. Su P, Wang Q, Bi E, Ma X, Liu L, Yang M, Qian J, Yi Q: **Enhanced Lipid Accumulation and Metabolism Are Required for the Differentiation and Activation of Tumor-Associated Macrophages**. *Cancer Res* 2020, **80**(7):1438-1450.

2. Liu L, Bi E, Ma X, Xiong W, Qian J, Ye L, Su P, Wang Q, Xiao L, Yang M *et al*: **Enhanced CAR-T activity against established tumors by polarizing human T cells to secrete interleukin-9**. *Nature communications* 2020, **11**(1):5902.

3. Yong J, Bischof H, Burgstaller S, Siirin M, Murphy A, Malli R, Kaufman RJ: **Mitochondria supply ATP to the ER through a mechanism antagonized by cytosolic Ca(2)**. *Elife* 2019, **8**.

4. Kanekura K, Ma X, Murphy JT, Zhu LJ, Diwan A, Urano F: **IRE1 prevents endoplasmic reticulum membrane permeabilization and cell death under pathological conditions**. *Sci Signal* 2015, **8**(382):ra62.

5. Kanekura K, Ishigaki S, Merksamer PI, Papa FR, Urano F: **Establishment of a system for monitoring endoplasmic reticulum redox state in mammalian cells**. *Lab Invest* 2013, **93**(11):1254-1258.
